# Centrilobular nodules with central calcifications

**DOI:** 10.36416/1806-3756/e20250021

**Published:** 2025-05-21

**Authors:** Edson Marchiori, Bruno Hochhegger, Gláucia Zanetti

**Affiliations:** 1. Universidade Federal do Rio de Janeiro, Rio de Janeiro (RJ) Brasil.; 2. University of Florida, Gainesville (FL) USA.

**Figure 1:**
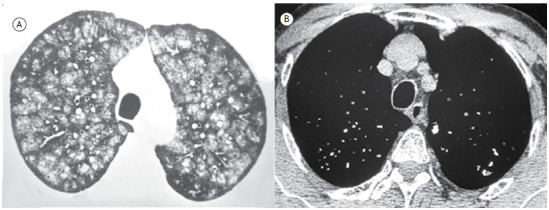
In A, axial CT scan of the chest with lung window setting at the level of the upper lobes shows nodular ground-glass opacities in a predominantly centrilobular distribution. In B, mediastinal window setting demonstrating small foci of calcification, corresponding to the ground-glass nodules.

A 67-year-old man complained of chronic cough and dyspnea on exertion. He had undergone renal transplantation for chronic renal failure many years ago. CT scans showed centrilobular nodular ground-glass opacities, predominantly in the upper lobes, some of which showed a central focus of calcification (Figure 1).

The nodules presented with a typical centrilobular distribution, sparing the pleural surfaces. The main diseases that present with this pattern are silicosis, hypersensitivity pneumonitis, some forms of bronchiolitis and metastatic pulmonary calcification (MPC). In most cases, the nodules of hypersensitivity pneumonitis and bronchiolitis have ground-glass attenuation. In hypersensitivity pneumonitis, a history of exposure to certain antigens generally aids in the diagnosis. In bronchiolitis, the nodules are often associated with the tree-in-bud pattern, which corresponds to the presence of centrilobular branching opacities, more evident in the lung periphery, resembling the budding appearance of some plants. The centrilobular nodules of this patient had unique characteristics that differentiated them from other diseases: they had ground-glass attenuation, imprecise contours, and central foci of calcification, making them practically pathognomonic of MPC.

CPM is a metabolic lung disease characterized by calcium deposition in normal lung tissue under conditions that directly or indirectly result in hypercalcemia. It most commonly arises as a long-term complication of chronic renal failure, especially seen in patients undergoing dialysis. Other causes include primary hyperparathyroidism, excessive exogenous administration of calcium and vitamin D, massive osteolysis due to metastases or multiple myeloma, orthotopic liver transplantation, and cardiac surgery, among others.^1^ ^,^ ^2^

Clinical manifestations of MPC are usually minimal; when present, symptoms are nonspecific and include dyspnea and nonproductive cough. Acute respiratory failure has been described, although it is rare. Therefore, the diagnosis is often initially suspected based on imaging findings. The relative stability of pulmonary infiltrates and their persistence despite treatment, in the presence of hypercalcemia, are important diagnostic clues. The main pattern observed on chest CT is nodular ground-glass opacities in the centrilobular airspace, with or without intervening foci of calcification. Other findings include high-attenuation consolidations (dense consolidation), calcification in vessels of the chest wall, and pleural effusion. The changes are usually bilateral.^1^ ^,^ ^2^ The patient underwent lung biopsy, which showed thickening of the alveolar septa due to fibrosis, in addition to calcium deposits in their walls, confirming the diagnosis of MPC.
